# Structural Aspects Affecting Phase Selection in Inorganic Zeolite Synthesis

**DOI:** 10.1021/acs.chemmater.2c03204

**Published:** 2022-11-22

**Authors:** Karel Asselman, Dries Vandenabeele, Nick Pellens, Nikolaus Doppelhammer, Christine E.A. Kirschhock, Eric Breynaert

**Affiliations:** † Center for Surface Chemistry and Catalysis—Characterisation and Application Team (COK-KAT), KU Leuven, Celestijnenlaan 200F, Leuven3000, Belgium; ‡ Institute for Microelectronics and Microsystems, JKU Linz, Linz4040, Austria; § NMR-Xray Platform for Convergence Research (NMRCoRe), KU Leuven, Celestijnenlaan 200F, Leuven3000, Belgium

## Abstract

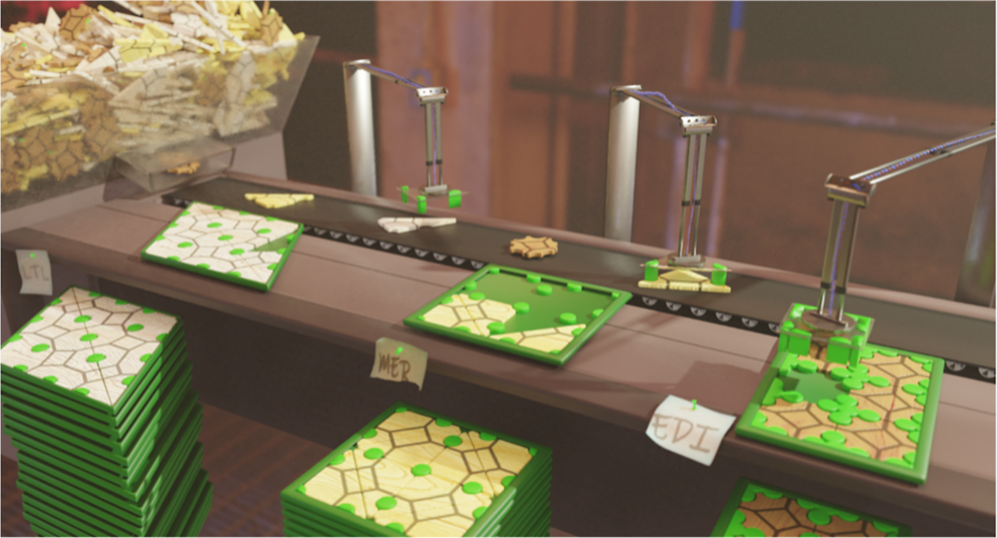

A guideline for zeolite phase selection in inorganic synthesis media is proposed, based on a systematic exploration of synthesis from inorganic media using liquid Na^+^, K^+^, and Cs^+^ aluminosilicate. Although the Si/Al ratio of the zeolites is a continuous function of the synthesis conditions, boundaries between topologies are sharp. The here-derived phase selection criterion relates the obtained zeolite topology to the Si/Al ratio imposed by the synthesis medium. For a given Si/Al ratio, the framework with the highest occupation of topologically available cation sites is favored. The large number of published zeolite syntheses supporting the observation provides strong indication that the concept is applicable in a larger context. The proposed criterion explains how minor variations in the composition of inorganic synthesis media induce the commonly occurring, abrupt changes in topology. It highlights underlying reasons causing the strict demarcation of stability fields of the as-synthesized zeolites experimentally observed in inorganic synthesis.

## 1 Introduction

Zeolites are porous members of the tectosilicate family, with numerous uses in catalysis, ion-exchange, or gas separations. Inorganic cations are known to play structure-directing roles in zeolite synthesis.^1−4^ Most inorganic cations template a range of aluminosilicate zeolites covering vastly different topologies and pore geometries. Small changes in batch stoichiometry can induce radical changes in phase selection, leading to crystallization of highly different and often structurally unrelated topologies. The rational connection between synthesis composition (type of cations, alkalinity, etc), framework topology, and aluminum content has however remained ambiguous.

Evaluating the thermochemistry of aluminosilicate zeolites isolated from their growth medium, isomorphic substitution of silicon by aluminum and an extra-framework cation Si^4+^ ↔ Al^3+^ + 1/*n* M^*n*+^ has been identified as a decisive factor governing the crystal energy of a zeolite.^5−9^ For a given cation, the enthalpy of formation, across different topologies, varies linearly with increasing Al content and corresponding cation content.^7,9^ This reflects the increasing Coulombic contribution to the crystal energy. Calorimetric studies revealed large cations with a lower charge density, that is, cations with a lower hydration energy, to stabilize zeolite frameworks more efficiently than small, hard cations. Structural studies demonstrated the former type to engage in a higher number of inner-sphere framework interactions.^7,10^ Comparing zeolites with the same Si/Al ratio and cation(s), the enthalpy of formation correlates with topological parameters such as framework density (FD), ring size, and so forth. The influence of these parameters is however small compared to the long-range Coulombic contributions reflecting the charge distribution and total cation content.^7^ These observations seem to suggest that aluminosilicate zeolites should invariably form with a Si/Al ratio of 1, maximizing Coulombic stabilization. This is in discord with many experimental inorganic zeolite syntheses, as frameworks with a Si/Al ratio exceeding unity are readily obtained. The discrepancy between the stability trends derived for aluminosilicate zeolites isolated from their synthesis media and the experimental phase selection of the as-made zeolites indicates that zeolite formation is determined by more than crystal energy alone.

To understand phase selection, zeolite stability must be evaluated with respect to the environment a zeolite resides in during formation, taking into account the overall free energy of the entire crystallization system in the assessment of the relative stabilities of zeolites during phase selection.^11,12^ A further complication is the participation of water in zeolite formation and its partitioning during crystallization. Most as-synthesized zeolites contain water, hinting at cation solvation in the synthesis medium versus coordination by framework and/or water in the crystal as a co-determining factor driving crystallization. At low synthesis temperatures, the enthalpy of hydration of extra-framework cations can enhance the crystal energy of porous frameworks with respect to anhydrous, denser polymorphs. At high synthesis temperatures, the unfavorable configurational entropy of hydration water confined in a zeolite framework can result in (re-)crystallization to a less hydrated material, typically exhibiting smaller cages.^9,12,13^

### 1.1 Aluminosilicate Speciation Governs Zeolite Solubility and the Resulting Framework Si/Al Ratio

The stability of any mineral with respect to the surrounding liquid can be expressed via a solubility product. For simple, ionic minerals such as salts, the solubility product is readily defined as the product of its individual ionic constituents. For minerals with covalent networks, such as aluminosilicate zeolites, solubility products are less easily defined. Consequently, since the exact nature of the soluble framework forming units is complex and the relevant thermodynamic speciation models for (alumino)silicate oligomers in alkaline solutions are largely unavailable, the stability of zeolites with respect to the liquid growth medium is difficult to evaluate.

A notable exception is the prediction of the LTA-FAU crystallization diagram by McCormick and Šefčík via development of a simple aluminosilicate speciation model in dilute, highly alkaline aluminosilicate solutions.^12,14^ Through definition of solubility products, they demonstrated that, depending on the stoichiometry of the mother liquor, zeolites with Si/Al > 1 can become stable in equilibrium with the surrounding synthesis solution, even though they would be metastable ex situ, that is, removed from the mother liquor, with respect to zeolites with higher aluminum contents.^7^ In other words, the solubility of aluminosilicate zeolites was shown to be a function of the framework Si/Al ratio. Zeolite stability and solubility in aluminosilicate synthesis media, consequently, are determined by the concentration and speciation of (alumino)silicate oligomers in these media. This speciation depends on the molar composition of the synthesis liquid and also on the type of alkali cation (infra). The approach used in the study was based on the assumption of (pseudo-)equilibrium between the final zeolite product and the supernatants. The accurate correspondence between the predicted and experimental crystallization diagrams demonstrated that phase behavior can be predicted based on solubility considerations alone, eliminating complex variables such as competitive nucleation or kinetics between different frameworks. It is therefore applicable to systems with extended crystallization and equilibration times, describing the long-term crystallization behavior, where the observed phase is not determined by competitive growth of different frameworks in early stages of synthesis but instead by the thermodynamic stability in the crystallization medium.^12^

Even though the solution model in the study could only be explicitly defined for a limited compositional range, the theoretical framework provided is general. It can, in principle, be extended to any molar composition of the mother liquor, provided accurate knowledge of aluminosilicate speciation and stability constants in a broad compositional range becomes available.^12,14^

The expression of solubility products however only includes the product of compositional units of the crystallizing mineral but implies in itself no specific framework topology. The underlying reasons for topology selection in inorganic zeolite synthesis thus remain unresolved.

The strong link between the composition of the synthesis liquid and the resulting framework Si/Al ratio was further demonstrated by Lechert and co-workers, who disclosed empirical relations between the synthesis batch stoichiometry and the framework Si/Al ratio of the formed zeolite product. For a number of topologies, the framework Si/Al ratio was shown to be a linear function of the batch alkalinity expressed as [SiO_2_]/[OH^–^].^15−18^

Recently, crystallization of aluminosilicate zeolites from monophasic liquids based on hypohydrated, aluminum-doped hydrated silicate ionic liquids (HSILs) was studied.^1^ In these systems, zeolites crystallize from homogeneous, true liquids. Total aluminum contents in the precursor liquids and resulting solid yield are very low, and all aluminate is found as soluble, aluminosilicate oligomers of low nuclearity in the precursor solution.^1,19−21^ A study of growth kinetics^22^ revealed that fast dynamics in the HSILs ensure that the growing crystal surface is always in contact with a homogeneous growth medium wherein the solubility of the aluminosilicate growth units with respect to the forming framework determines the progress of solid yield. With long incubation times of 7 days, synthesis can be expected to run to completion. Therefore, as an approximation, the assumption of pseudo-equilibrium between the supernatant solution and final zeolite products (supra) holds true for these systems. It follows that thermodynamic considerations, such as solubility and crystal energy, can be used to rationalize the phase behavior in these systems (i.e., framework topology and composition), separately from competitive nucleation and crystallization kinetics.

## 2 Methods

**Figure 1:**
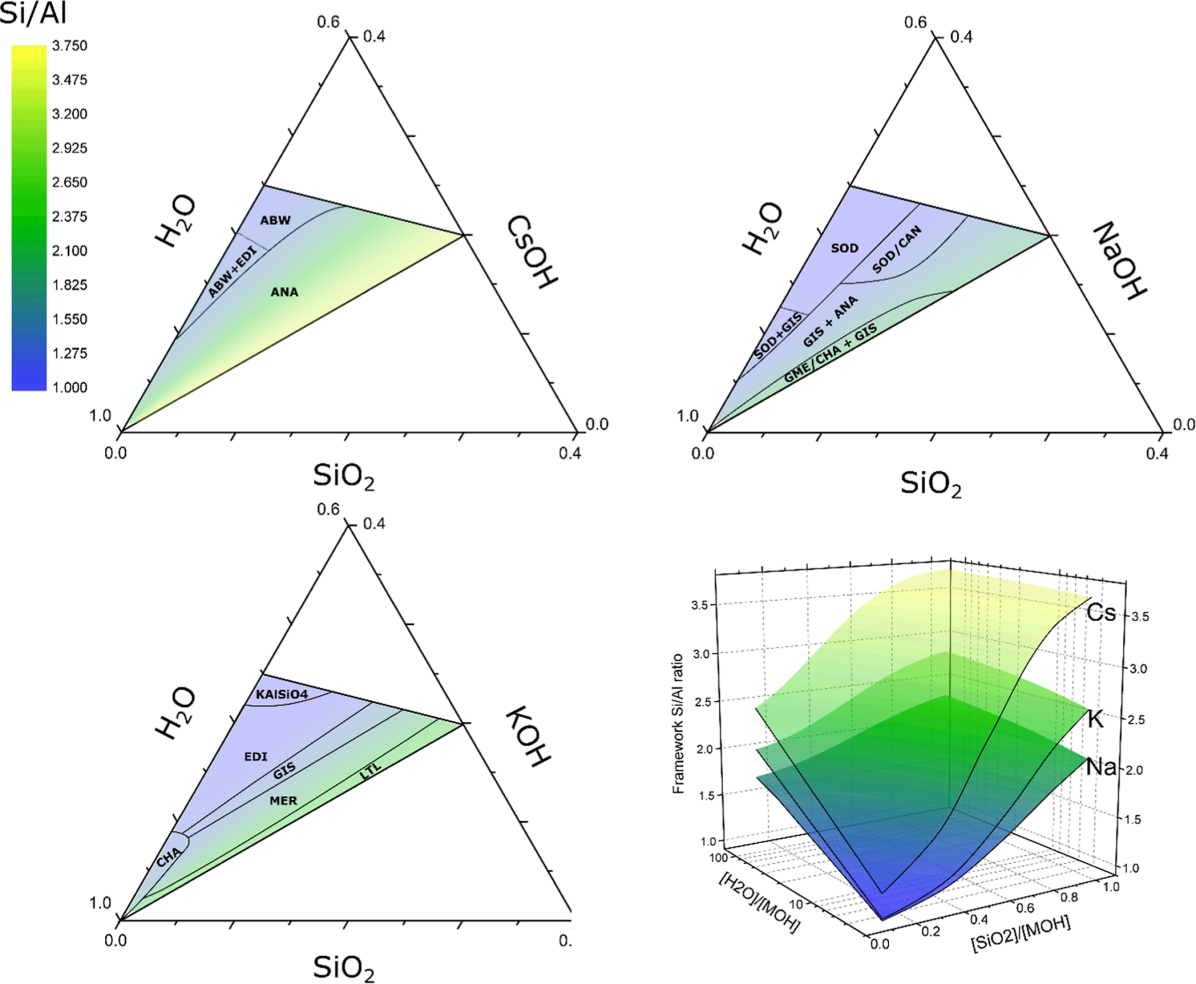
Ternary phase diagram representation of synthesis data from ref (1) displaying phase boundaries in the 0.5 SiO_2_/0.013 Al_2_O_3_/*x* MOH/*y* H_2_O chemical space synthesized at 90 °C and framework Si/Al ratio values of synthesis products as a function of batch alkalinity [SiO_2_]/[MOH] and cation hydration [H_2_O]/[MOH].

HSILs are hypohydrated alkali-silicate solutions, formed through spontaneous coacervation and phase separation in TEOS–H_2_O–MOH mixtures (M = Na, K, and Cs). The native HSIL is combined with appropriate amounts of water and alkali hydroxide, and doped with aluminate, to achieve desired synthesis mixtures with molar composition 0.5 SiO_2_/0.013 Al_2_O_3_/*x* MOH/*y* H_2_O. For the syntheses presented in Figure 1, samples were hydrothermally incubated for 7 days at 90 °C in a tumbling oven. For more details on the synthesis procedure and on compositions of the native HSIL and synthesis mixtures used, we refer the reader to the original publication.^1^

## 3 Results and Discussion

### 3.1 Observations Derived from Zeolite Synthesis in HSILs

Topologies and Al contents of zeolites obtained in that study^1^ are summarized in Figure 1, in ternary diagram representation. Eleven different topologies, including the most commonly occurring framework types in conventional, inorganic hydrothermal zeolite synthesis, were obtained. The Si/Al ratio of the crystallizing zeolites was shown to be a continuous, smooth function of batch alkalinity and water content of the synthesis liquid. Furthermore, a strong dependency of the alkali cation type on the resulting framework Si/Al ratio was revealed. This was attributed to the observed ion-pairing of aluminosilicate ions with the alkali cations, impacting their speciation.^1,20^ Gradual changes in the batch stoichiometry result in gradual changes in the relative abundance of the different aluminosilicate oligomers and consequently also in gradual changes in the average Si/Al ratio of the aluminosilicate oligomers in the liquid state. This gradual change is reflected in a gradual variation of the Si/Al ratio of the forming zeolites. In contrast, the phase boundaries separating zeolites with different topologies were observed to be quite abrupt, similar to crystallization diagrams reported in the literature for inorganic zeolite synthesis.^12,13,23,24^ This is best exemplified by the KOH-based phase diagram displayed in Figure 1. Variation of the batch alkalinity successively produces EDI–GIS–MER and LTL topologies. In parallel, the measured framework Si/Al ratio varies smoothly from 1 to approximately 3. Each topology forms within a well-defined and relatively narrow range of framework Si/Al values. The experimental data set from that study was supplemented with numerous observations in the literature on conventional hydrothermal zeolite synthesis, and typical ranges for framework Si/Al values for a respective topology and cation are listed in Table 1. These fields of stability, or existence, also depend on the cation type. For instance, in the ANA and GIS topologies, experimentally observed Si/Al ranges are different for different cations (Figure 1 and Table 1). This implies that the framework aluminum content and the cation type, both imposed by synthesis parameters, are major factors deciding phase selection. Apparently, for a given cation, the Si/Al ratio of the framework critically affects the efficiency of the mutual stabilization of cations and the framework in a given zeolite topology.

**Table 1:** Cation Sites Represented in the Highest Topological Symmetry and Projected Minimum Si/Al Ratio of Zeolite Frameworks, with Comparison to Experimental Values from Natural or Synthetically Produced Zeolites Reported in the Literature^a^

framework	M^+^	TO_2_/uc	cation sites	Mult.	M^+^/uc (max)	Si/Al (min)	exp. range^c^	references
LTL	K	36	t-can	2	11	2.3	2.3–3.5	(43), (62)–^65^
			t-kaa	3				
			t-ste ∩ t-lil	6				
MER	K	32	t-pau ∩ t-opr	4	12	1.7	1.7–2.3	(23), (53), (66),^67^
			t-ste ∩ t-pau	8				
GIS	K	16	t-gsm ∩ t-gsm	8	8	1.0	1.2–1.4^d^	(1)(68)–^72^
	Na						1.0–3.4	
EDI	K	5	t-kdt	2	3	0.7^b^	1.0–1.2	(1), (24), (46)
			t-krq	1				
CHA	K	36	t-cha ∩ t-hpr	6	15	1.4	1.4–2.7	(2)(73)–^76^
			t-cha ∩ t-cha	9				
kalsilite	K	4		2	2	1.0	1.0	(1), (77)
ANA	K	48	t-ana	16	16	2	∼2	(1), (34), (78), (79)
	Cs						∼2–4^e^	
	Na		t-kds	24	24	1	1.5–3.10	
GME	Na	24	t-gme	4	10	1.4	∼2	(80)–^82^
			t-gme ∩ t-kno	6				
SOD	Na	12	t-toc	8	8	0.5^b^	1.0	(47), (83)
CAN	Na	12	t-can ∩ t-can	2	8	0.5^b^	1.0	(49), (84), (85)
ABW	Cs	8	t-kdq	4	4	1	1–1.3	(1), (28), (86), (87)

a Cation sites have been indicated with their corresponding natural tiles. A “∩” symbol indicates that the cation is located in the window separating two adjacent tiles.

b Pores include anions at high cation occupancy, avoiding violation of Löwensteins rule.

c This range only includes frameworks formed in homo-ionic systems without the presence of alternative structure-directing agents or salts, that is, only alkali hydroxides.

d Aluminosilicate K-GIS has so far only been observed in our studies;^1^ therefore, the effective range of possible experimental Si/Al values may be larger for this material.

e Pollucite is usually encountered with ideal stoichiometry (Si/Al = 2). In our studies, pollucites were produced hydrothermally with a Si/Al ratio of up to 4.

In the following paragraphs, we first highlight some common relations between extra-framework cations and zeolite topologies. Then, we develop novel insights into the framework composition–topology relation and the crucial role of the extra-framework cation in phase selection.

### 3.2 Common Observations on the Interaction between Framework Topology and Extra-framework Cations

During crystallization, the affinity of cations for either framework interaction or hydration exerts a pivotal role in phase selection. Following crystallization, cations reside on or close to specific, cation-dependent sites in the zeolite structure.^25^ In the as-made zeolites, cations are typically found in a state of optimal coordination by framework oxygen and/or hydration water. With increasing softness, cation coordination to framework oxygen often is preferred over coordination to water. Cation positions are thus modulated by the cations’ enthalpy of hydration. The lower the enthalpy of hydration of the cation, the stronger the affinity of the cation for direct framework coordination and vice versa. This observation applies in a larger context, as it also determines the selectivity in monovalent ion-exchange in clays.^26^ It readily explains why alkali cations with the lowest hydration energy more frequently reside in strongly bound inner-sphere ion-exchange positions. In analogy, alkali cations show preference for typical environments within a zeolite. For instance, Na^+^ often resides in front of six-membered aluminosilicate rings (6R), forming a sixfold octahedral coordination with three framework oxygens and three water molecules.^25^ K^+^ shows a rather clear preference for eight-membered rings (8R), with a lower, but still notable, affinity for water, which often shields cations on neighboring positions. Cs^+^, the most polarizable, stable alkali cation, is most often confined in zeolite cavities where it coordinates to eight or more framework oxygens, but rarely to water.^27,28^

**Figure 2:**
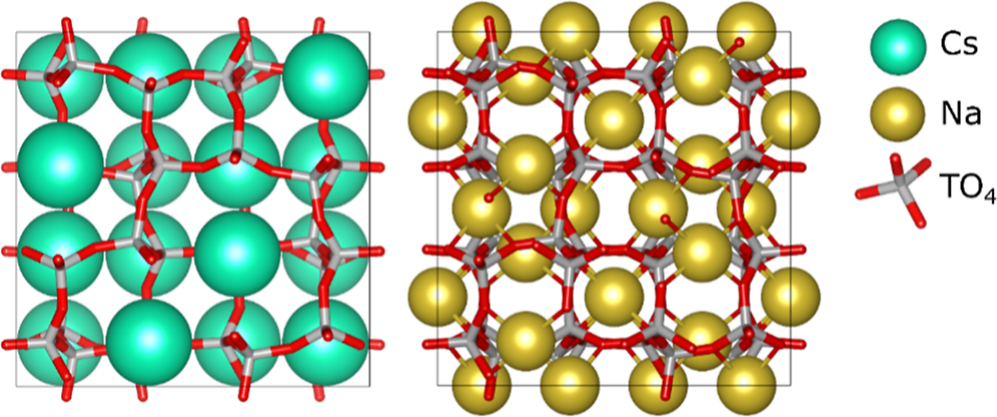
Structure of Cs-ANA (left) and Na-ANA (right) (hydration water not shown). Cs and Na occupy distinct sites in the framework (crystal ionic radii of cations drawn to size^61^). At full cation occupancy, both minerals would have respective Si/Al ratios of 2 and 1. The structure of K-ANA or leucite is analogous to pollucite.

A zeolite topology formed in the presence of alkali cations offers distinct favorable topological cation sites for the available cation(s). Vice versa, the preference of cations for specific coordination environments causes many aluminosilicate zeolite topologies to exclusively form in the presence of specific inorganic cations (e.g., Na-SOD, Na-FAU, K-LTL, ...). Some framework topologies present different sites specific for different cations. This allows these frameworks to crystallize with different alkali cations (e.g., EDI with K^+^ or Cs^+^, ANA with Na^+^, K^+^, or Cs^+^, and MER with K^+^ or Rb^+^, ...). Some cases even require, or are favored by, the simultaneous presence of two cation types (e.g., JBW with Na^+^ and K^+^,^29^ PHI with Na^+^ and K^+^,^30^ and RHO with Na^+^ and Cs^+^^31^). The naturally occurring ANA topology provides an instructive example (Figure 2). In K^+^-ANA (mineral/leucite) and Cs^+^-ANA (mineral/pollucite), cations are located in the large cavity (A-site).^32^ In Na^+^-ANA (mineral/analcime), Na^+^, a smaller cation, resides in the small window connecting two adjacent A-cavities (S-site), where it also coordinates to water, located between neighboring cations.^33^ Occupation of adjacent A- and S-sites are mutually exclusive due to their proximity. This leads to specific cation-ordering schemes for Cs-pollucites, partially substituted by Na^+^.^34^

A distinction needs to be made between the as-synthesized and post-synthetically modified zeolites. Extra-framework cations in zeolites often can be readily replaced by a range of other cations, a modification greatly impacting the stability of the material.^9,35^ For example, replacing monovalent alkali cations by earth-alkali provides options to increase thermal resilience^36^ by stronger framework coordination. This however simultaneously reduces thermodynamic stability, due to increased charge gradients, and/or increased strain on bonding angles of the aluminosilicate frame.^7,37^ Likewise, ion-exchange with the very hard, strongly polarizing Li^+^ cation can induce a significantly reduced thermal stability^7,38−40^ and can even lead to (partial) framework failure.^41^ Combined, these observations clearly indicate zeolite stability to be determined by a fine balance between optimum cation coordination and favorable framework stabilization.

### 3.3 Connection between Framework Topology and Framework Si/Al Ratio

As outlined in the Introduction section, the final Si/Al ratio of a crystallizing zeolite topology is determined by the composition of the synthesis medium and the cation type, through considerations of solubility. Phase selection must also be linked to the same parameters. It should thus be possible to rationalize phase selection and explain why a specific topology is preferred for a specific framework composition.

The first important aspect in predicting the outcome of a synthesis is the topological cation capacity of a zeolite framework. The topological cation capacity of a zeolite framework for a specific cation can be defined as the maximum number of preferred coordination positions for that cation, known from crystallography. When all these cation sites are occupied, a framework can be considered saturated. This topological cation capacity defines the minimal Si/Al ratio of a defect-free zeolite framework crystallizing in the presence of a specific cation. A cation-saturated framework with maximal framework aluminum content, that is, the minimal Si/Al ratio, is defined by
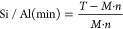
1with *T* and *M* representing the total number of T-sites and the maximal number of viable sites for cations with valence *n+* per unit cell, respectively. In a cation-saturated framework, the crystal energy is maximized by optimization of framework–cation interactions and by minimizing charge gradients. At the same time, all cations are found in positions optimally coordinated by framework oxygen and, if present, hydration water. Equation 1 assumes that all negative framework charge arises from framework aluminum. Other systematic sources of negative framework charge, such as periodic anionic lattice defects, are negligible for inorganic, high-alumina frameworks crystallizing in purely inorganic media (see discussion in the Supporting Information).

**Figure 3:**
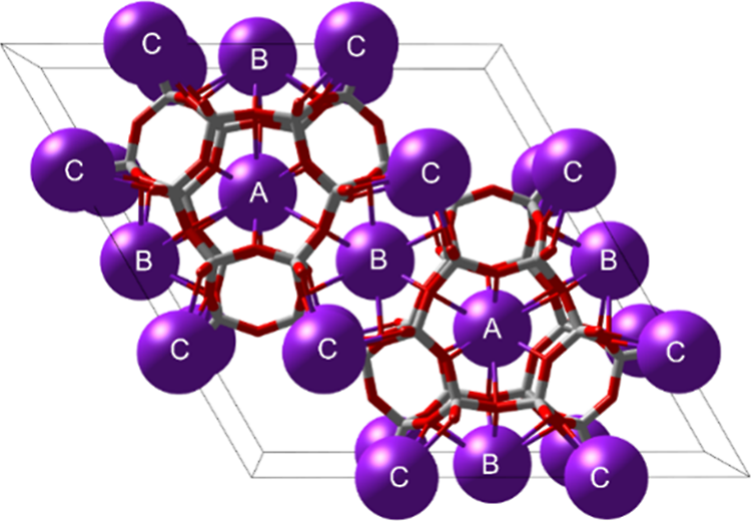
Representation of distinct crystallographic K sites (A–C) in LTL topology (crystal ionic radii of cations drawn to size^61^).

Using this definition, a minimal Si/Al ratio can be derived from the crystal structure of any homo-ionic, as-synthesized framework. In the case of K—LTL, it has experimentally and computationally been established that K^+^ is preferentially occupying three distinct crystallographic sites.^42,43^ The first site (A-site) is located in the cancrinite cage (Figure 3), the second site (B-site) resides in the central channel connecting neighboring cancrinite cages, and the third site (C-site) is found in the nonplanar 8-ring at the periphery of the 12-ring channel. Although K^+^ has been observed on additional sites, close or even inside the double six-rings on top or below the cancrinite cage, these sites are much less selective and, due to proximity, cannot be occupied simultaneously with site A. Occupation of such sites hence does not affect the maximum cation capacity. In the high symmetry description of the LTL topology (space group *P*6/mmm), the A-, B-, and C- sites have multiplicities of 2, 3, and 6, respectively, bringing the maximum number of cations to 11 K^+^ per unit cell. One unit cell in the LTL framework contains 36 T-sites; therefore, the theoretical minimal Si/Al ratio of K-LTL is
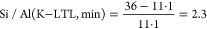


Si/Al = 2.3 is indeed experimentally observed to be the minimal Si/Al ratio for K-LTL, both in the systematic crystallization study supporting this work (Figure 1) and also in the literature (Table 1). Note that introducing divalent cations in the synthesis provides a way to increase the Al content and thus lower the Si/Al ratio. K^+^ and Ba^2+^ exhibit similar ionic radii and prefer similar sites. The simultaneous presence of K^+^ and Ba^2+^ during LTL crystallization enforces one additional framework Al-site for each additional Ba^2+^ ion incorporated in the unit cell with respect to a homo-ionic K^+^-based synthesis. In a K^+^- and Ba^2+^-containing synthesis, LTL has indeed been synthesized with Si/Al = 1, the highest possible Al content not violating the Löwenstein rule.^44^ In analogy to the example of K-LTL, minimal Si/Al ratios for a number of common homo-ionic frameworks have been derived and are listed in Table 1. The corresponding frameworks and cation distributions are visualized in the Supporting Information (Figure S1–S13). For all listed topologies, preferential cation sites were derived from published crystallographic data of the as-synthesized zeolites. For simplicity and clarity, Table 1 and Figures S1–S13 list the topologies in the highest symmetry space group with the associated cation sites positioned on high symmetry sites (special positions). Although zeolite materials quite often exhibit lower space groups and/or cation displacement from the high symmetry sites, such deviations from the high symmetry situation do not affect the maximal possible cation content per unit cell.

Some entries in Table 1 warrant more detailed explanation, as they represent didactic examples with important implications for phase selection. As indicated in eq 1, the minimal Si/Al ratio of a topology is cation-dependent. Analcime provides again the showcase example supporting this statement. In the ANA topology, the number of favorable sites per unit cell is different for Na^+^ and K^+^ or Cs^+^ as the multiplicities of their preferred sites are different. Na^+^ preferentially occupies the S-site with multiplicity 24, while K and Cs preferentially reside in the A-site, occurring with multiplicity 16. Consequently, the ANA topology can maximally accommodate 24 Na^+^ but only 16 K^+^ or Cs^+^ ions. With 48 T-atoms per unit cell, the lower Si/Al limit for K^+^- and Cs^+^-ANA is strictly 2, while Na^+^-ANA can occur with a higher Al content (Table 1 and Figure 2). Special situations are found in the SOD, CAN, and EDI topologies. Unlike other topologies, these frameworks offer more topological cation sites than can be charge-compensated by framework charges without violating the Löwenstein rule.^45^ With complete topological cation occupancies, corresponding to Si/Al ratios of 0.75, 0.75, and 0.83 in Na-SOD, Na-CAN, and K-EDI respectively (Figures S6, S12, and 13), full topological cation occupancy would require an Al content with a Si/Al ratio below 1, which is never observed experimentally. Peculiarly, these frameworks still achieve full topological cation occupancy, via co-inclusion of extra-framework anions (OH^–^, Cl^–^, CO_3_^2–^, ...). This prevents violation of the Löwenstein rule, while maintaining charge balance and maximizing the number of cations included in the structure.^46−49^ Hydroxysodalite is the best-known example.^47,50^ It crystallizes in ultra-alkaline solutions with a Si/Na^+^ ratio in the framework lower than 1. If all charges would be compensated by the framework, the Si/Al ratio would drop below one. Inclusion of extra-framework hydroxide allows crystallizing the framework with a Si/Al of 1 and a cation content exceeding the framework charge. By removing the hydroxysodalite crystals from their synthesis medium and making them to come into contact with water, the extra-framework cation–anion pairs are easily and irreversibly removed from the zeolite.^50^ It was demonstrated that hydroxysodalite only forms under synthesis conditions where Na^+^-OH^–^ ion pairs are already stabilized in the solution to allow simultaneous sodium-hydroxide inclusion: ultra-alkaline, concentrated synthesis liquids providing sufficiently high hydroxide activity.^50^

In contrast to the definition of a lower Si/Al boundary (eq 1) of a topology, which is strict and cation-dependent, the upper boundary is not limited in a straightforward fashion. In principle, the Si/Al ratio could, in theory, extend up to infinity for a purely siliceous framework. With increasing Si/Al ratio however, increasingly larger parts of the framework will become less occupied and less stabilized by cations, thus generating an increasing deviation from the ideal charge distribution. This is unfavorable for the crystal energy of the forming solid. While some of the framework stabilization might be compensated by water clusters, the increasing hydrophobicity of regions with increasingly siliceous character also limits this option.^51^

**Figure 4:**
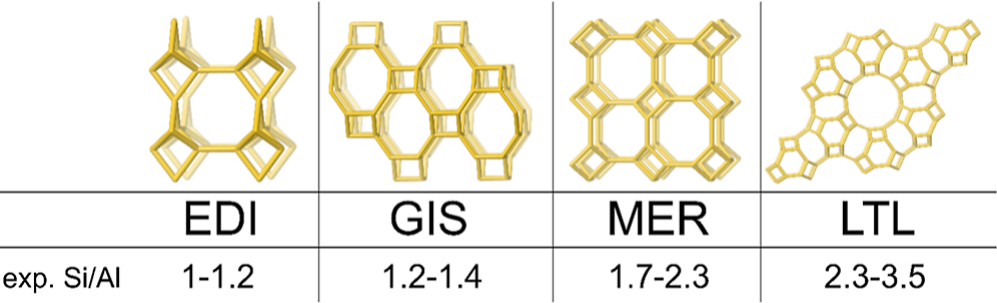
Experimentally observed Si/Al ratios for some frameworks with adjacent crystallization fields in KOH-based synthesis mixtures.

**Figure 5:**
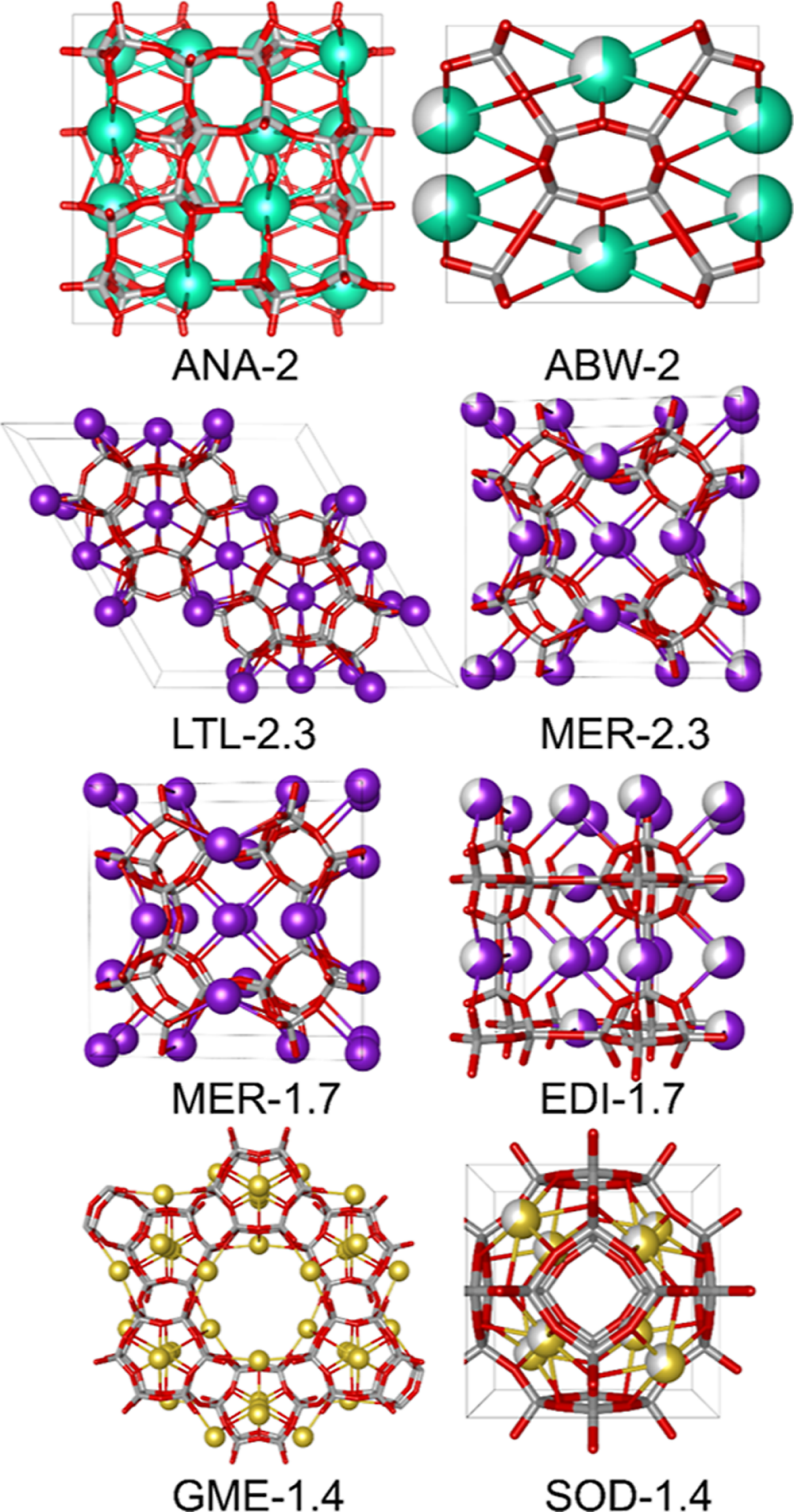
Comparison of relative cation vacancies between some Cs- (green), K- (violet), and Na (yellow)-based topologies with identical stoichiometries (ions not drawn to size). Numbers indicate hypothetical framework Si/Al ratios. For identical compositions, different frameworks display different average cation occupancy numbers due to varying number of viable cation positions. During synthesis, the framework with the highest occupancy number of available cation positions is favored, especially for larger, softer cations.

Experimentally, we establish that the upper boundary of the Si/Al ratio is often defined by the lower boundary of the neighboring topology in the ternary diagram. In a systematic homo-ionic synthesis series crystallizing multiple topologies from varying batch compositions, the topology with the highest occupation of topologically available cation sites is preferred at a given framework Si/Al ratio. This is best illustrated by the K- and Cs-based synthesis system (Figures 1, 4, and 5). Lowering the alkalinity ([SiO_2_]/[KOH]) of the synthesis mixture, the topology of the crystallizing zeolites changes quite abruptly at specific Si/Al ratios.^1^ With increasing Si/Al ratio, sharp phase boundaries exist between EDI, GIS, MER, and LTL topologies, with phase mixtures only observed close to the phase boundaries.^1^ As K-LTL is the end of the studied series, no upper boundary can be projected for this framework. K-MER exists with a framework Si/Al ratio ranging from 1.7, that is, its projected lower limit (using eq 1), up to 2.3, coinciding with the lower limit of K-LTL (Figure 4). At Si/Al = 2.3, crystallization of K-LTL with a fractional topological cation occupancy of 1, the maximum possible, is apparently preferred over crystallization of K-MER with a fractional topological cation occupancy of 0.81 (Figure 5). In KOH-based syntheses crystallizing zeolites with Si/Al ≥ 2.3, K-LTL is always favored over K-MER, as apparent from the wider literature. As elaborated earlier, for synthesis mixtures crystallizing zeolites with Si/Al < 2.3, K-LTL crystallization is prevented as this framework does not provide sufficient topological cation sites (supra) to balance the negative framework charge resulting at this Al content. In analogy, a maximal Si/Al ratio of 1.7 is implicated for K-GIS, coinciding with the lowest possible Si/Al ratio for K-MER. At this Si/Al ratio, the fractional topological cation occupancy is 1 for K-MER and 0.75 for K-GIS.

As elaborated earlier, K-EDI is a special topology with similarities to hydroxysodalite, which crystallizes in the exact same compositional region of the Na-ternary diagram (Figure 1). Indeed, like hydroxysodalite, K-EDI crystallizes with OH^–^ anions occluded in its cages.^46^ At these high batch alkalinities and limited water contents, hydroxide activity is high enough to stabilize hydroxide-cation ion pairs in the synthesis medium.^50,52^ K-EDI formation is preferred under these conditions over K-GIS only if sufficient alkalinity allows inclusion of “excess” cations via co-inclusion of extra-framework hydroxide, optimizing total cation–framework interaction and Coulomb energy. This option does not apply to K-GIS, as its available cation positions are already fully occupied at a Si/Al ratio of 1. As the batch alkalinity ([SiO_2_]/[KOH]) decreases, hydroxide inclusion is prevented as hydroxide activity is reduced, and a phase change to K-GIS is observed in our studies at Si/Al ≈ 1.2. K-GIS has a higher fractional cation occupancy, that is, less cation vacancies, than K-EDI when they have identical stoichiometries (Table 1), so this phase change is consistent with subsequent phase changes in K-MER and K-LTL (supra). In the Na-based diagram (Figure 1), an analogous transition is observed: as a result of extra-framework hydroxide incorporation, under ultra-alkaline conditions, crystallization of (hydroxy-)SOD prevails over crystallization of Na- GIS or Na-ANA.

The same considerations readily apply to the Cs-ternary diagram. Cs-EDI and Cs-ABW, two topologies with a suitable number of topological cation sites to form zeolites with Si/Al = 1, crystallize at the highest batch alkalinities. With increasing framework Si/Al ratio, induced by decreasing alkalinity or increasing dilution, a phase change to Cs-ANA is triggered at Si/Al = 2. In the ANA topology, Cs ions are located on a single crystallographic site. At Si/Al = 2, this Cs site is fully occupied, and Cs-ANA achieves a cation-saturated state. In a hypothetical Cs-ABW with a Si/Al ratio of 2, one-third of all topological Cs sites would have to be vacant to respect charge neutrality (Figure 5).

From these observations, it can be postulated that frameworks with a given Si/Al ratio and cation content minimize their energy by adopting a topology with the highest fractional occupancy of the topologically available cation sites. In a fully occupied state, framework coordination and stabilization by the cation are optimal, and charge distribution and gradients are homogeneous and minimal. Conversely, with increasing number of cation vacancies, cation–framework interaction energy is reduced while the charge distribution (cations and Al-sites) becomes increasingly inhomogeneous, reducing lattice energy.

### 3.4 Maximal Cation Coordination to Framework Oxygen Stabilizes Zeolite Frameworks

As discussed, as-synthesized K-MER exists with a framework Si/Al ratio ranging from 1.7 up to 2.3. While at Si/Al ratio of 1.7, all topological cation sites are 100% occupied, increasing its Si/Al ratio requires MER to crystallize with a fractional topological cation occupancy <1. In this evolution, K-MER has been observed to retain near full occupancy in the cation sites most strongly coordinated to the framework, that is, the sites located in the window between t-pau and t-ste tiles, exhibiting a coordination number (CN) of 8 (Figure S4). Vacant topological cation sites occur preferentially in sites near the D8R, a cation position with lower coordination to the framework (CN = 4).^23,53^ Similarly, in K-LTL with a Si/Al ratio ≥2.3, unoccupied topological cation sites are always found in the 12R-pore, while the anhydrous cation sites in and in between the cancrinite cages, with maximal CN = 12 to framework oxygen, remain fully occupied.^43,54^ This emphasizes the importance of cation coordination by framework oxygen for zeolite stabilization. When synthesis conditions enforce the formation of an unsaturated zeolite with unoccupied topological cation sites, the structure is stabilized as much as possible by selectively omitting cations from the sites least coordinated by the framework.

Zeolites are dense upon genesis, the voids in their topology being filled and stabilized in an optimal way by solvent and template molecules. Organic templates select for high Si/Al topologies offering optimal stabilization by maximizing interaction with the zeolite framework without introducing large amounts of charge. This is typically achieved through some shape resemblance between the organic and the pore geometry. For zeolites templated by inorganic cations, the Si/Al ratio is limited by the size, charge, and coordination chemistry of the inorganic cations. Inorganic cations are comparatively small, and many of them are required to maximize interaction with the framework oxygen. At the same time, every cation requires charge compensation, inherently limiting the maximum Si/Al ratio that can be achieved in purely inorganic synthesis, unless cation–anion pairs can be co-included into the framework. Eventually, the number of extra-framework cations becomes too limited to effectively stabilize the zeolite framework, thus limiting the maximal Si/Al ratio that can be achieved within the system. As the Si/Al ratio of a purely inorganic zeolite framework increases, topologies with fewer topological cation sites are adopted, while each individual cation site offers more framework coordination partners, thus maximizing the interaction per cation. This is visualized in Figure S14 for topologies observed in the K- and Cs-systems. Perhaps counterintuitively, this inevitably results in generation of larger pores in newly formed topologies. EDI, GIS, MER, and LTL exhibit highly similar overall framework densities, ranging from 16.3 to 16.7 T-sites/1000 Å. Interestingly, LTL, with the highest Si/Al in the series, has the largest pore diameter: 12-membered rings (12R) versus 8Rs for the other topologies. The presence of wider pores, without decreasing the overall FD, implies coexistence of denser framework regions next to less dense ones (i.e., wider pores). In K-LTL, dense sections of interconnected pillars of alternating cancrinite cages and D6R separate adjacent 12MR pores (Figures 3–5). At Si/Al = 2.3, the K-LTL framework is fully “saturated” with potassium. Five cations are found in fully anhydrous sections of the framework inside and in between the cancrinite cages. These sections are dense, and their cations coordinate to up to 12 framework oxygen on sites A and B (Figure 3). The remaining six cations are lining the 12R pore, each interacting with six framework oxygens and completing their coordination shell with H_2_O molecules in the 12R channel. Coordination of all cations by the framework is maximized, and all sections of the framework are in close interaction with the cations, with every framework oxygen closely coordinating to at least one cation. In a MER zeolite with the same Si/Al ratio, the fractional topological cation occupancy would be significantly lower (0.81), leaving many vacant cation sites and framework oxygen not in contact with a nearby cation. Adopting a topology containing dense, small-pore alkali-aluminosilicate regions alternating with large pores, filled with a dynamic network of partially hydrated cations and liquid-like water,^42^ allows us to maximize cation–framework interactions. The combination of a fully occupied framework with high Coulombic contribution to the crystal energy, arising from the inner-sphere ion-pairing between the fully occupied framework and its cations, and with the entropic gain resulting from the participation of liquid-like water in a dynamic cation-water network in the large channels clearly outperforms situations with a lower cation occupancy where more isolated water is contained in smaller pores or cages. As a result, K-LTL gains stability over a partially occupied K-MER even though the former contains the large 12R-pores.

### 3.5 Competition between Cation Coordination by Either Framework Atoms or Hydration Water

The proposed heuristic of maximizing cation occupancy is based solely on cation–framework coordination energy and does not yet explicitly account for the role of water and hydration energy. As demonstrated, it works well for almost all phase transitions in the K- and Cs-ternary diagrams since these are large cations exhibiting a high affinity for framework oxygen with respect to their comparatively low affinity for water.^7,9,55−57^ In edge cases however, the basic criterion falls short, requiring to also account for the role of water as a coordination partner for the cations explicitly.

The Na-based phase diagram is more complex than the cases of Cs and K (Figure 1). Na-zeolite synthesis is more subject to phase mixtures, intergrowths, recrystallization over time, and temperature, while spanning a smaller range of possible framework Si/Al ratios.^1,13^ The spectrum of Na-polymorphism is broader, with more frameworks having overlapping ranges of possible framework compositions compared to K- and Cs-zeolites (Table 1). Still, phase transitions from SOD (6-rings) to GIS or ANA (8-rings) and eventually GME (12-rings) with decreasing framework Al content are observed for Na-based zeolites (Figure 1 and Table 1). Once again, these transitions correspond to the formation of frameworks with higher topological sodium occupancy, with increased sequestration of dense framework regions and larger pores (supra) as the framework Si/Al ratio increases. However, phase transitions do not always occur exactly on their expected value like in the K- and Cs-system. For instance, based strictly on the proposed heuristic, a phase change from GIS to GME is expected when the Si/Al ratio reaches a value of 1.4. Instead, phase transition to GME is only observed for Si/Al = 2 or higher. A plausible explanation for the added complexity is the lower framework–cation interaction energy compared to the hydration energy of the comparably hard sodium cation.^55−58^ Consequently, the selection criterion put forward in this manuscript may be less stringent for Na-based frameworks. The competition between water and the framework to respectively solvate the cation will be much stronger and may more explicitly contribute to phase selection. Topologies with a more favorable hydration state of the cation especially at low to moderate synthesis temperatures can be expected. It is important to note that phases occurring in mixtures in the ternary diagram (Figure 1), for example, GIS-ANA biphasic region, or as intergrowths, for example, GME/CHA, have an identical fractional cation occupancy at any given Si/Al ratio, that is, they offer an equal amount of coordination sites for sodium (Table 1) but differ in the room available for cation coordinating water molecules.

A similar consideration is also applicable for the larger cations, especially in the most water-deprived mixtures, where the few available water molecules are strongly held by the abundantly present cations. Framework selection along synthesis sequences with increasing dilution but constant alkalinity needs to account for the increasing number of water molecules available for cation coordination in the liquid. In the KOH-system, for example, phase transition from dense KAlSiO_4_ polymorphs to EDI and eventually CHA is observed in a dilution series of synthesis mixtures with a low but constant Si/KOH (Figure 1). Dense, anhydrous KAlSiO_4_ forms in the region where water content is minimal and aluminosilicate deprotonation is maximal (([H_2_O]/[KOH])_synthesis_ < 4 and ([KOH]/[SiO_2_]_synthesis_ > 8). It always has a Si/Al ratio of 1 and a fractional topological occupancy of 1. The formation of anhydrous KAlSiO4 polymorphs over hydrated EDI can be attributed to an extremely low water activity in these synthesis mixtures, apparently preventing inclusion of enough extra-framework hydroxide for K-EDI where cations also require hydration water,^46^ to gain stability over anhydrous KAlSiO_4_. These synthesis mixtures are essentially hyper-concentrated KOH solutions, with low amounts of framework-forming species. With less than four water molecules per cation, cations are hypohydrated, and all water molecules are locked in their first coordination sphere. Generally, hydration enthalpy provides a thermodynamic driving force for the formation of hydrated, porous aluminosilicate zeolites with respect to their dense, anhydrous counterparts and free, liquid water.^7,9^ No liquid water is however present in these hyper-concentrated systems, with every water molecule already functioning as hydration water for the cations in the solution. It is reasonable that the driving force promoting hydrated, porous frameworks is absent in these situations, resulting in the formation of dense KAlSiO_4_ polymorphs instead. Further dilution of the synthesis mixture promotes the formation of hydrated EDI and gradually decreases the charge density of the liquid and increases the Si/Al ratio in the products, eventually leading to chabazite for dilute samples when Si/Al reaches 1.4 (Figure 1 and Table 1), in accordance with the proposed selection criterion.

### 3.6 Implications for Zeolite Synthesis

Topology and Al content are the most important parameters defining the properties and function of a zeolite. This work proposes an explanation rationalizing why the Si/Al ratio of alkali zeolites is usually limited to the lower ranges. The insights provided here may however offer opportunities to extend the Si/Al ratio beyond their typical ranges. The example of using divalent cations to increase Al content of LTL down to a Si/Al ratio of 1 is one example and inclusion of anions, as observed in sodalite or edingtonite, is another.

The rationale that it is favorable for a framework to maximize the cation occupancy, combined with the observation that some frameworks spontaneously incorporate extra-framework anions to accept more cations than required to balance the framework charge, suggests a pathway toward higher silica materials synthesized fully inorganically. If extra-framework species could be introduced into the zeolite as cation–anion pairs, the framework can benefit from stabilization by the cation at a lower aluminum content. These ion pairs can be removed post-synthesis, simply by making the crystals to come into contact with water.^50^ Feasibility of this concept has very recently been demonstrated in the inorganic synthesis of KFI-type zeolite with unusually high silica content, by inclusion of K^+^–NO_3_^–^ pairs.^59^ The framework Si/Al ratio was 4.8, cation occupancy was found to be 100%, and the material remained stable after extraction of the excess ions. An earlier study^60^ describing the synthesis of high silica KFI (Si/Al = 5.2–5.8) also required the use of high concentrations of KNO_3_ in the synthesis batch as a source of potassium to obtain the desired product. In that study, however, it was not disclosed whether nitrate was co-included in the pores.

## 4 Conclusions and Outlook

A rationalization of the different zeolite topologies obtained in inorganic zeolite synthesis with similar compositions has been proposed. It was demonstrated that, for a given framework Si/Al ratio, phase selection favors a topology with the highest occupation number of viable cation sites. With decreasing framework aluminum content, framework structures allowing cations to coordinate to a larger number of framework oxygen emerge. This ensures efficient mutual stabilization of aluminosilicate and cations. The proposed heuristic works well for many zeolites where the cation shows a low affinity for interaction with water and prefers interaction with (alumino)silicate. Affinity for other coordination partners, such as anions or hydration water, was discussed as additional guidelines for rationalization of zeolite synthesis.

Although the framework Si/Al ratio of the forming zeolite can be estimated from the composition of the synthesis liquid based on explicit^12,14^ or empirical^1,15,17,18^ models, the heuristic reported in this paper holds potential as an important predictor for the obtained zeolite topology. Further development will require more detailed understanding of the speciation of aluminosilicate and cation-coordination in a broad spectrum of synthesis media. This work clearly shows that the crystallizing system needs to be evaluated as a whole to rationalize and predict the synthesis outcome. The stability of the forming crystal with respect to the physicochemical state of the medium determines the Si/Al ratio, as well as the resulting topology.

## Supporting Information Available

Illustration of cation distributions in all discussed topologies and visualization of cation coordination polyhedra and coordination numbers (PDF)
