# Supplementary material for: Structural Aspects Affecting Phase Selection in Inorganic Zeolite Synthesis
Source: Chem Mater. 2022 Nov 22;34(24):11081–92. doi: 10.1021/acs.chemmater.2c03204 (PMC9798827; doi:10.1021/acs.chemmater.2c03204)
Supplement: Supplementary file 1 — cm2c03204_si_001.pdf [file cm2c03204_si_001.pdf]

Supporting information to:

Structural aspects affecting phase selection in inorganic zeolite synthesis

Karel Asselman[1], Dries Vandenabeele[1], Nick Pellens[1], Nikolaus Doppelhammer[1,2], Christine E. A. Kirschhock[1,#] and Eric Breynaert[1,3,*,#]

[1]Center for Surface Chemistry and Catalysis – Characterization and Application Team (COK-KAT), KU Leuven, 3001 Leuven, Belgium

[2]Institute for Microelectronics and Microsystems, JKU Linz, Linz 4040, Austria

[3]NMR-Xray platform for Convergence Research (NMRCoRe), KU Leuven, 3001 Leuven, Belgium

*Corresponding author: eric.breynaert@kuleuven.be

#shared last author

On the calculation of minimal framework Si/Al ratio

1. Source of negative framework charge

The maximum number of cations that can be occluded in a framework depends on the number of topologically viable cation sites, and is therefore independent of the source of negative charges balancing their positive charge. While many zeolite samples may contain defects as result of a non-ideal crystallisation process, with slow, careful crystallization allows to synthesize all aluminosilicate zeolite topologies to be crystallized with a negligible concentration of defects, as also indicated by the chemical formula's of all reference materials listed in the IZA database for topologies discussed in this manuscript. Equation 1 in the main article, estimating the theoretically minimal framework Si/Al ratio, consequently assumes that all negative charge of the framework arises from framework aluminum sites, and not from anionic lattice defects. Exceptions of course confirm the rule, and for frameworks such as sodalite and edingtonite, where the concentration of framework Al not necessarily matches the concentration of extraframework cationic charges, inclusion of extra framework anions compensating the excess positive charge was demonstrated. The close, and often exact, correspondence between calculated minimal topological Si/Al ratio (using equation 1), and the minimal Si/Al ratio experimentally observed in our study and reported in literature, implies that the assumption that all negative framework charges arise from framework aluminum is valid.

For special cases such as high-silica (Si/Al > 10) or pure-silica materials synthesized with organic cationic templates, like SSZ-74 (-SVR) [1], YNU-2 (MSE) [2], systematic anionic lattice defects, can however be required in charge-balancing the extra-framework template. To our knowledge, such systematic incorporation of anionic framework defects (i.e., without post-synthetic modifications like dealumination) is limited to few, high-silica topologies and they are always synthesized in presence of (cationic) organic templates. For materials exclusively synthesized with inorganic cations, those discussed in the manuscript, we cannot find reports on systematic inclusion of anionic lattice defects upon genesis.

2. Symmetry representation of zeolite structures

Frameworks are represented in their highest topological symmetries for simplicity and clarity. In most structures, the experimentally observed symmetry is substantially lower due to distortion of the framework by its inherent flexibility, or even due to specific cation and framework aluminum ordering schemes. This should not affect the plausible cation positions with respect to the topology unless a strong local distortion renders a specific cation site unfavorable.

Cations are placed on crystallographically highly symmetrical sites (special positions) for all frameworks In reality, it is quite often observed, that cations are slightly displaced from their high symmetry site. For instance in K-chabazite, potassium is displaced from the center of the 8R towards the cavity (t-cha cage). Similarly, the Na cations for high-aluminum gismondine are shifted from the 8R center closer to its edges, optimizing framework-cation interaction. In both cases the result is a splitting of the cation site in two or more symmetrically equivalent sites close to the respective high-symmetry position. However, these sites are mutually exclusive due to their proximity and the sum of their occupation numbers is therefore limited to 1 per topologically symmetric site and representation in this way does not affect the absolute number of cation positions in a framework.

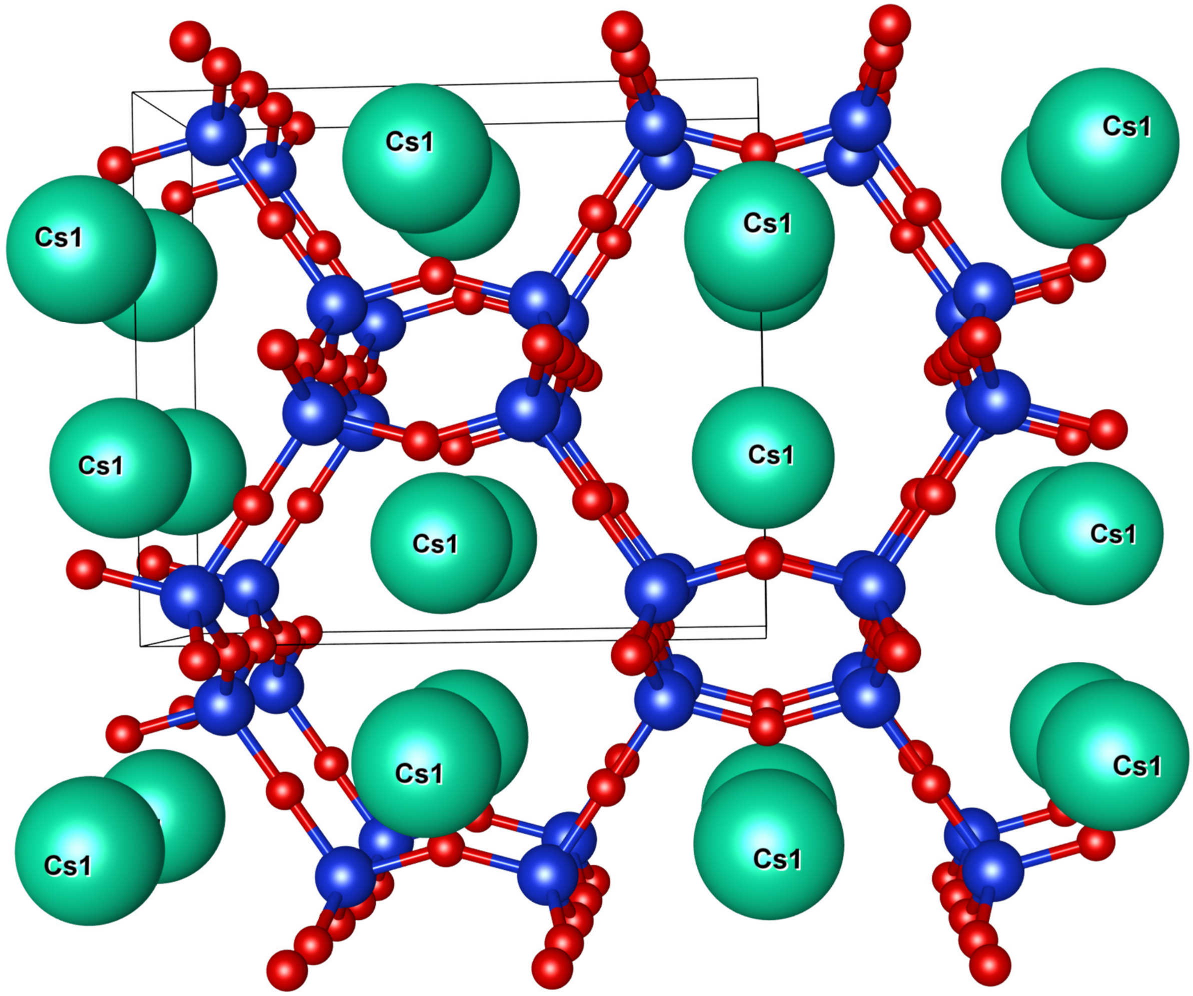

1. Cs-ABW (min Si/Al = 1)

All images were created in VESTA[3]*.*

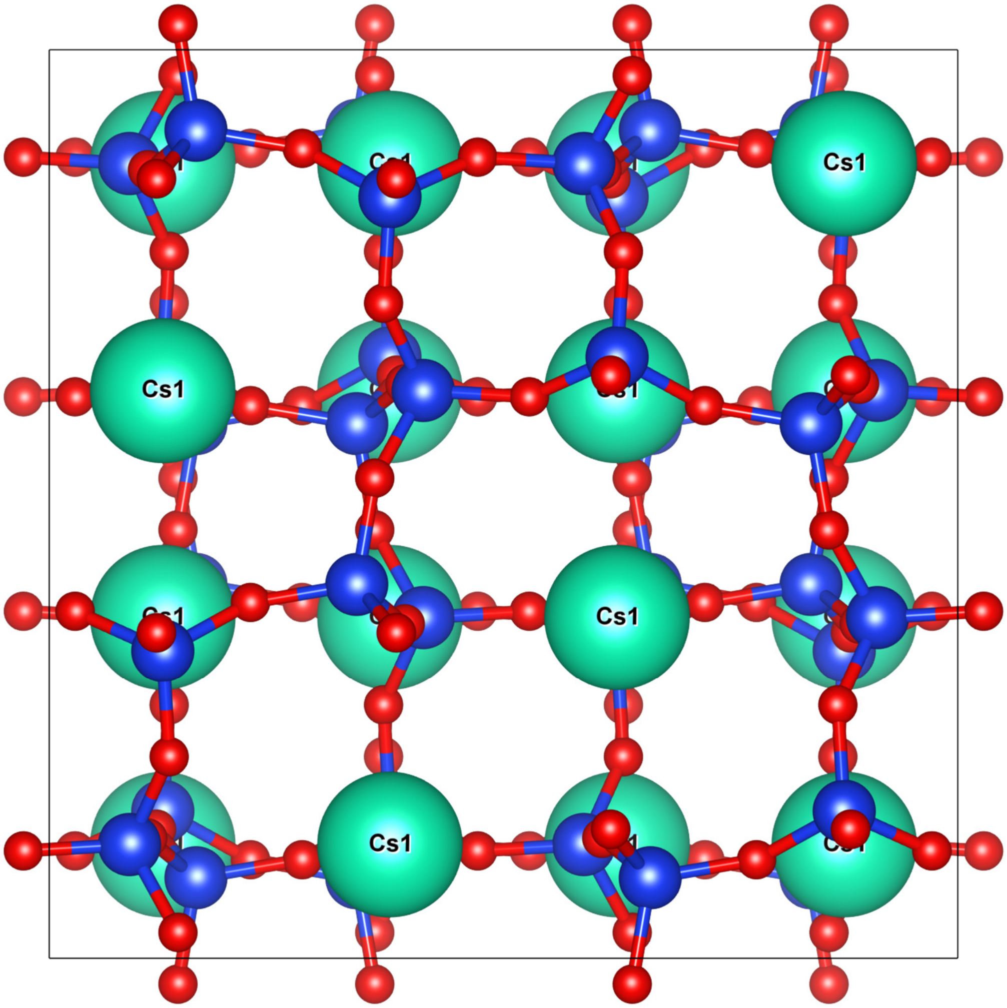

2. Cs-ANA (min Si/Al = 2)

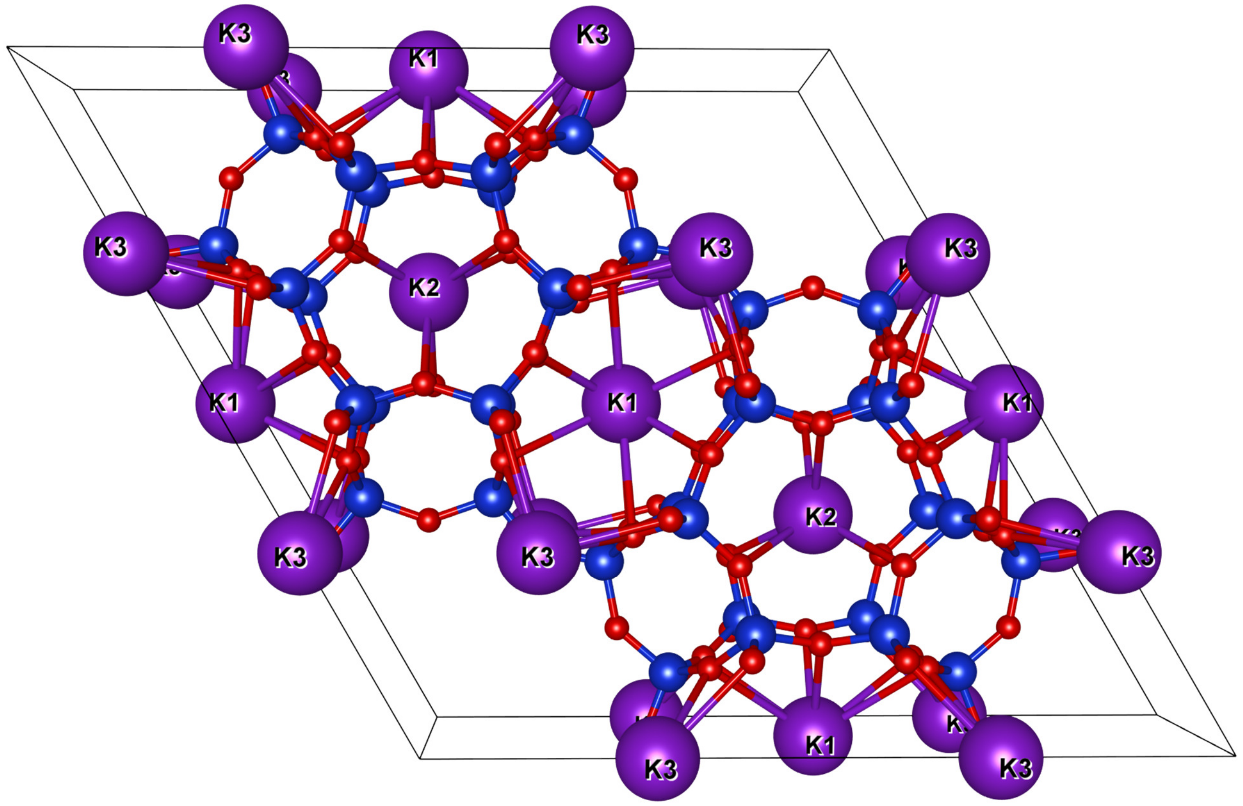

3. K-LTL (min Si/Al = 2.3)

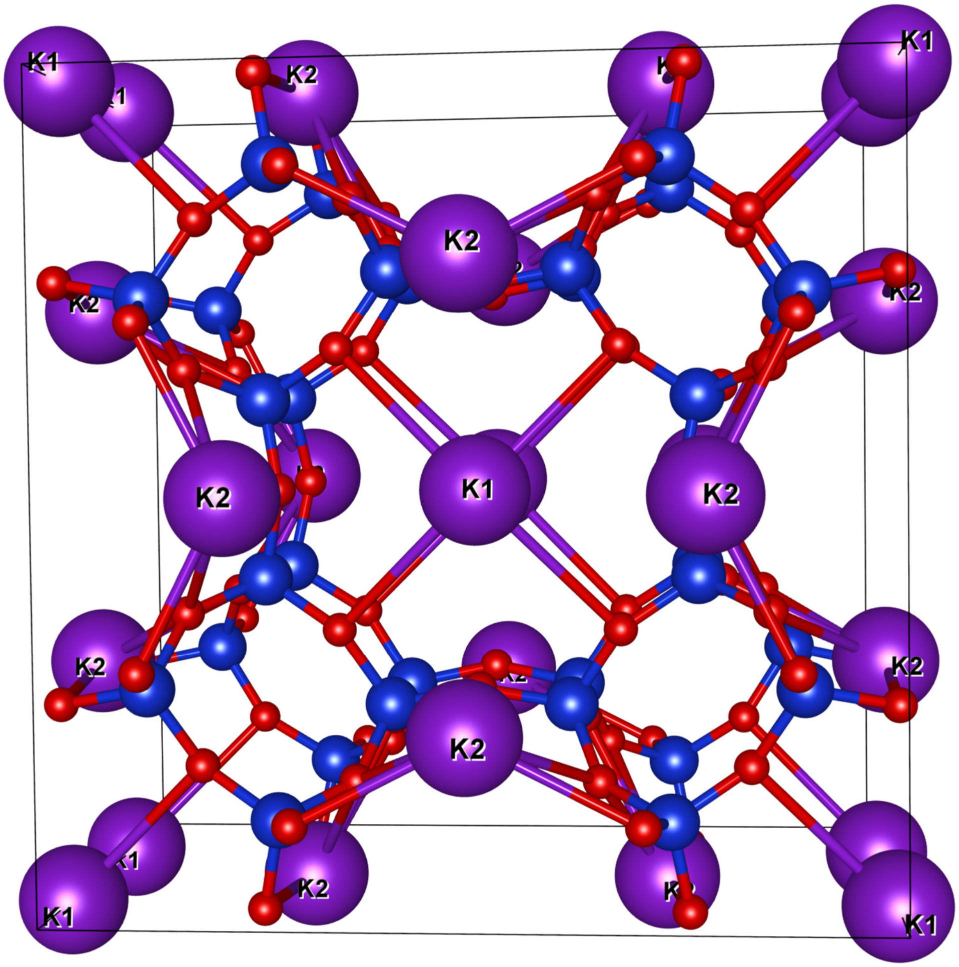

4. K-MER (min Si/Al = 1.7)

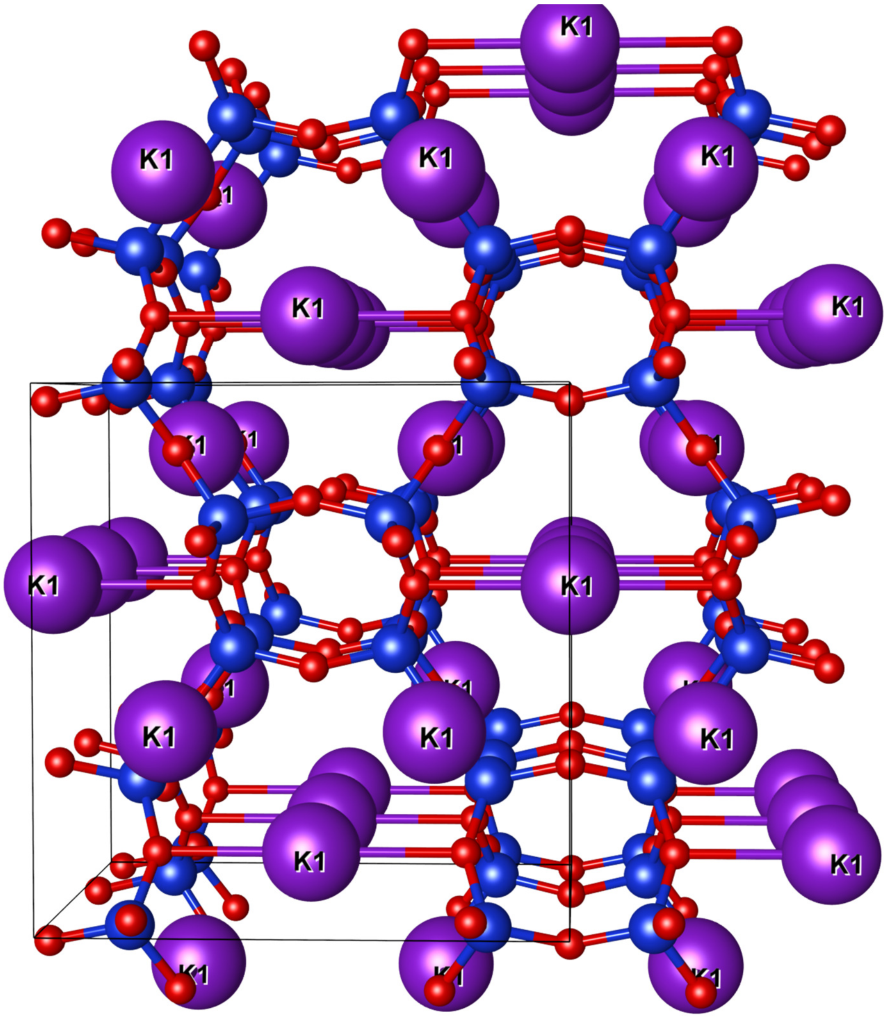

5. K-GIS (min Si/Al = 1)

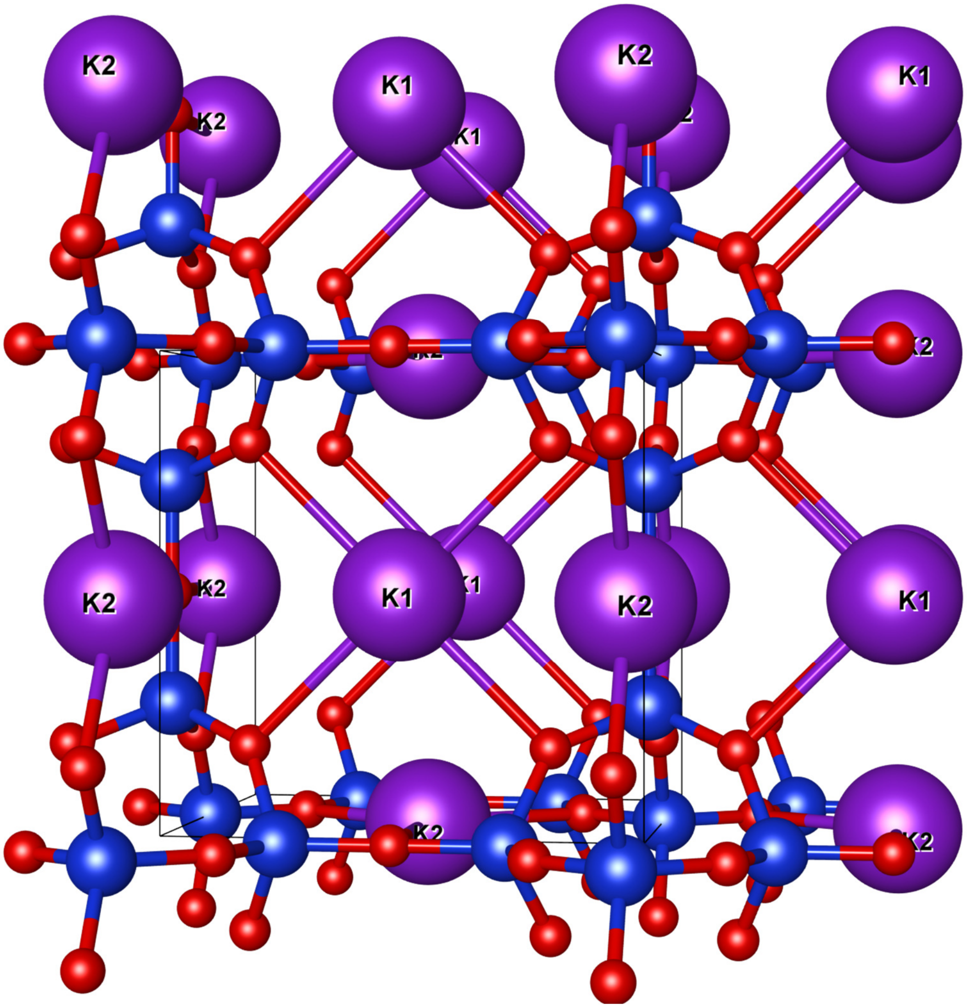

6. K-EDI (min Si/Al = 0.7*)

* EDI, SOD and CAN topologies allow inclusion of excess cations, and include anion to avoid violation of Lowenstein's rule.

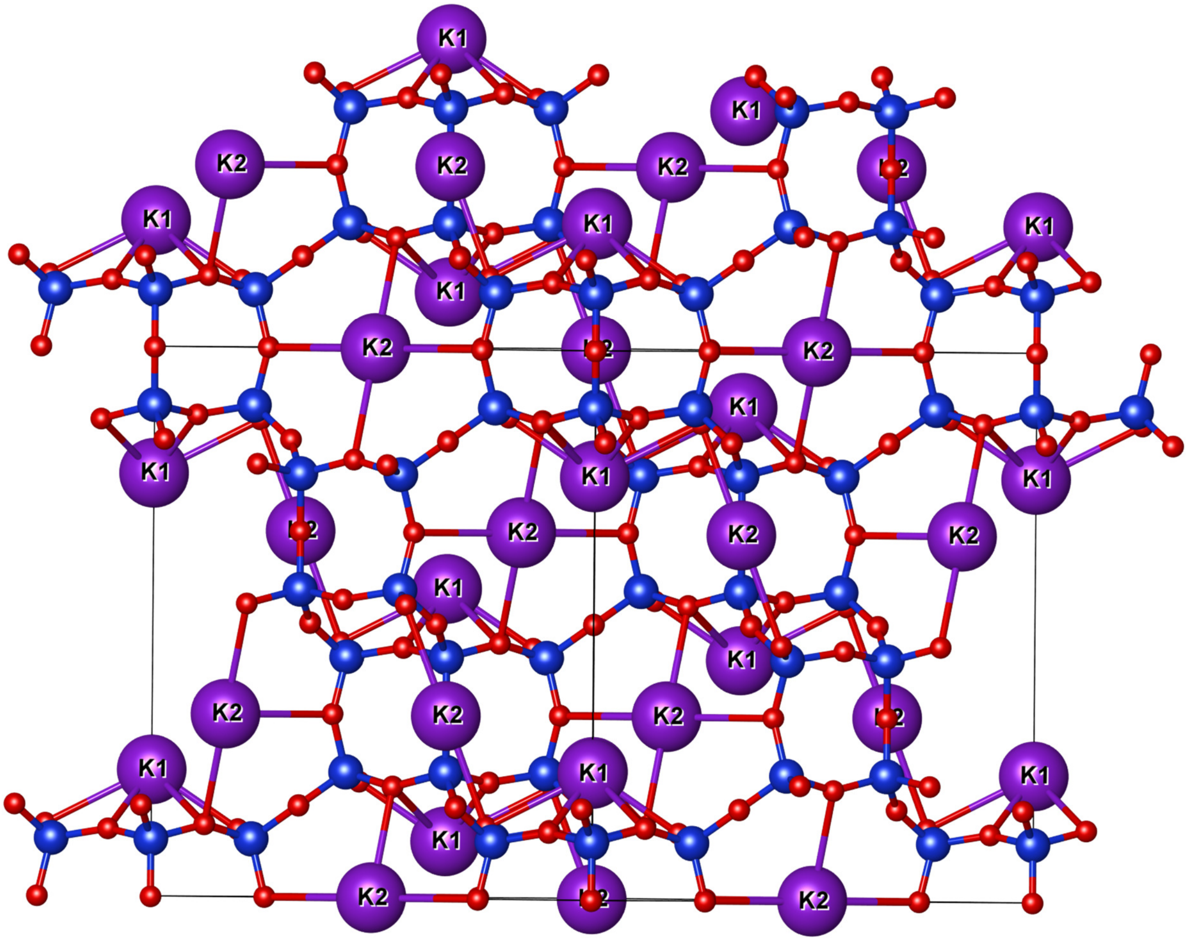

7. K-CHA (min Si/Al = 1.4)

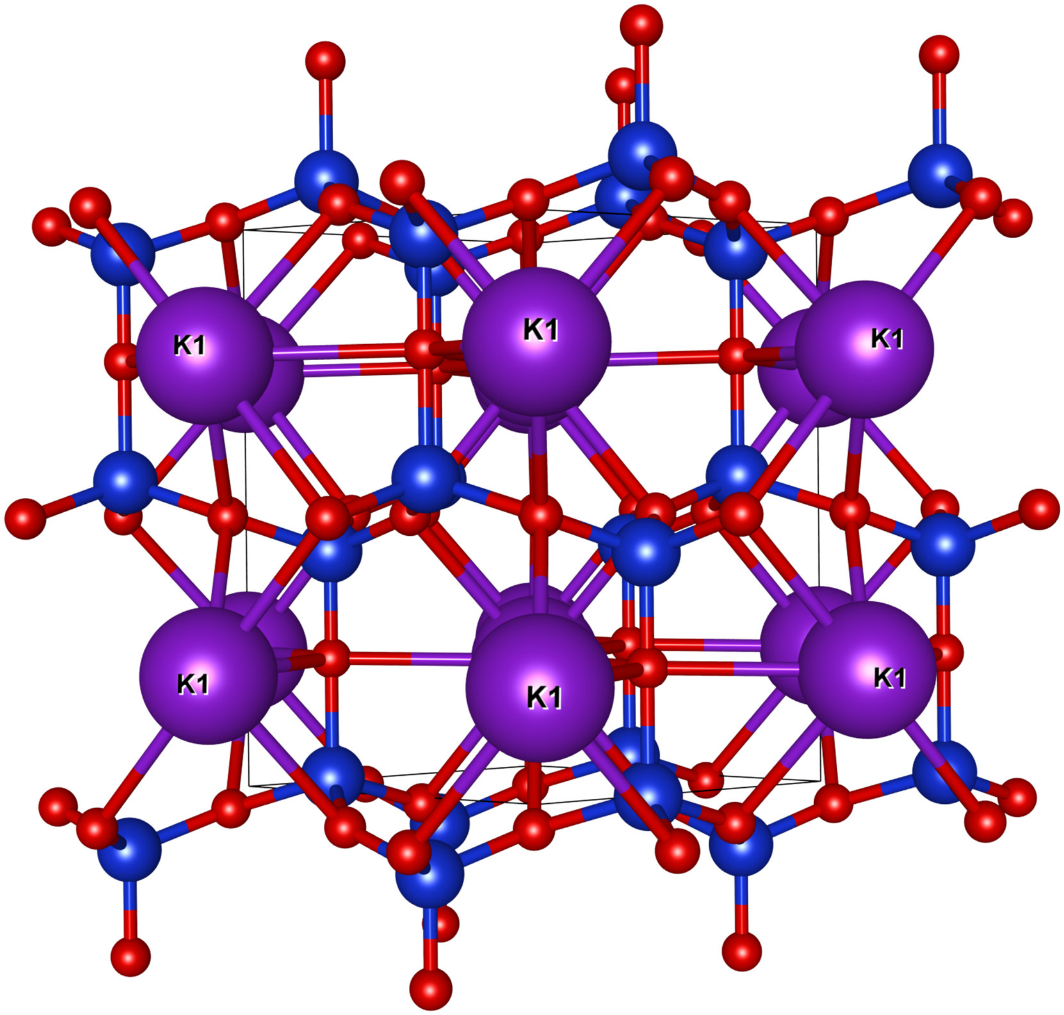

8. $KAlSiO_4$ (min Si/Al = 1.0)

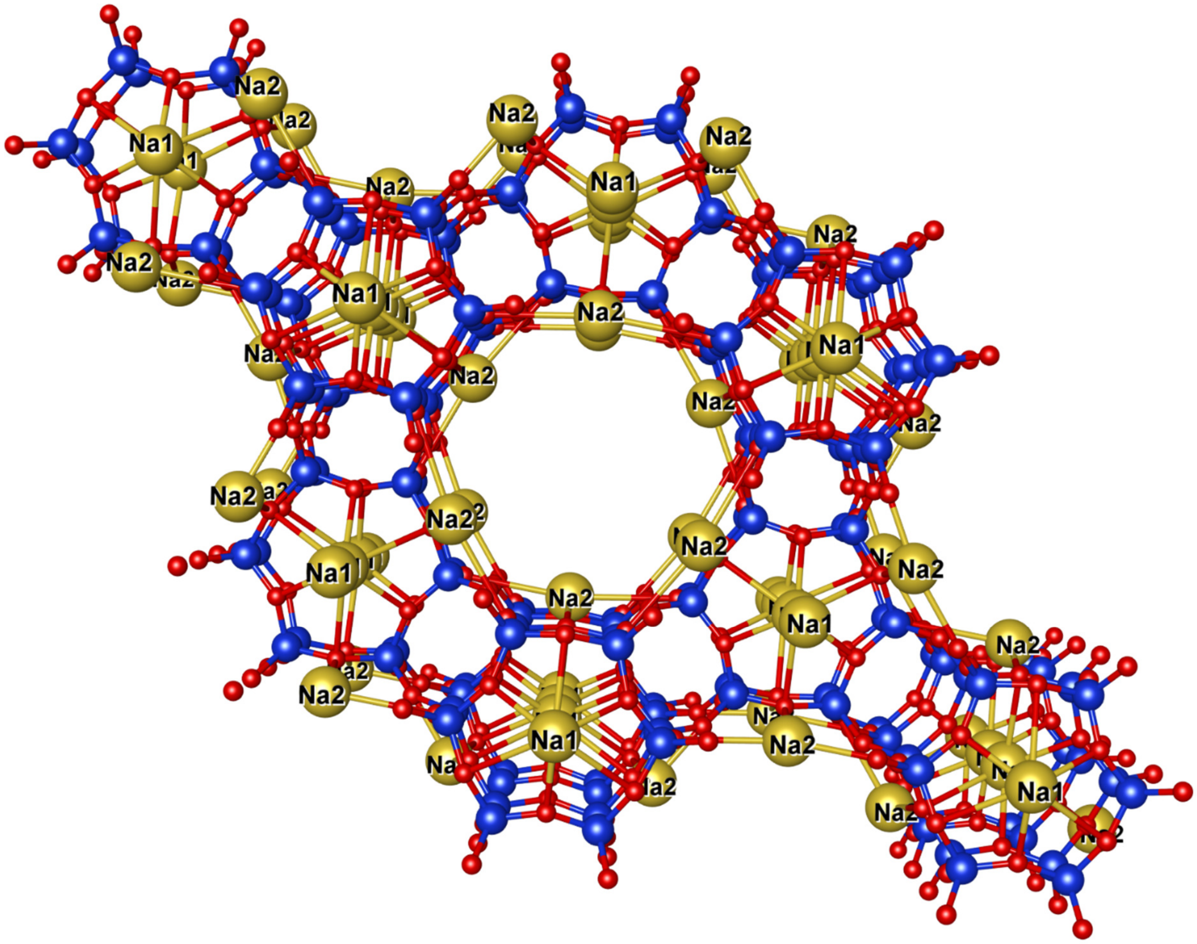

9. Na-GME (min Si/Al = 1.4)

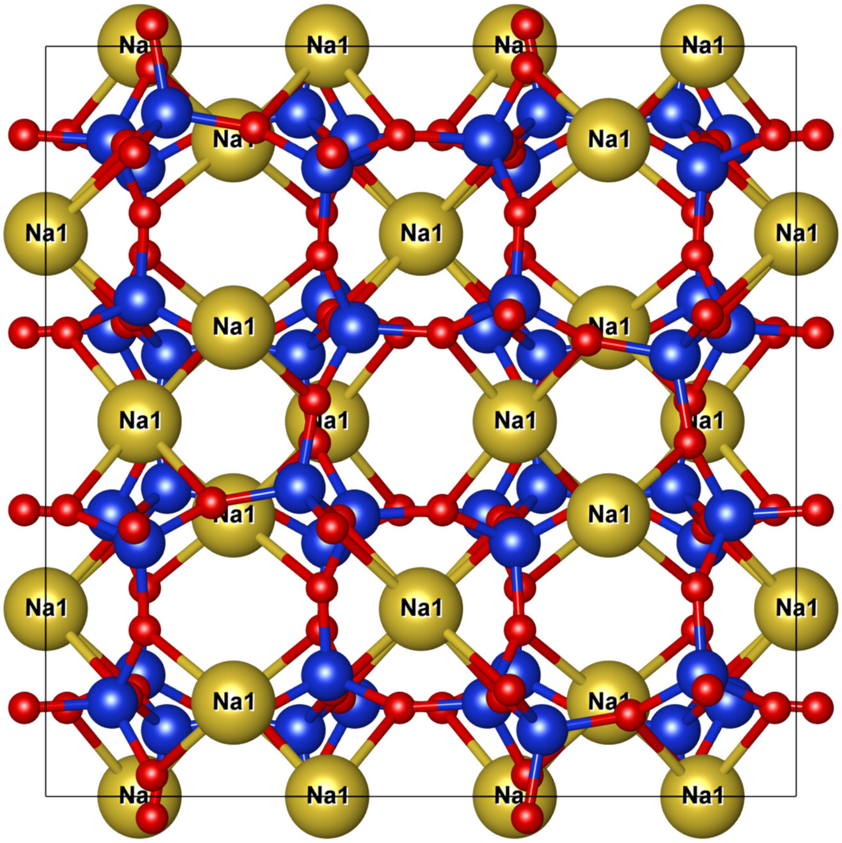

10. Na-ANA (min Si/Al = 1)

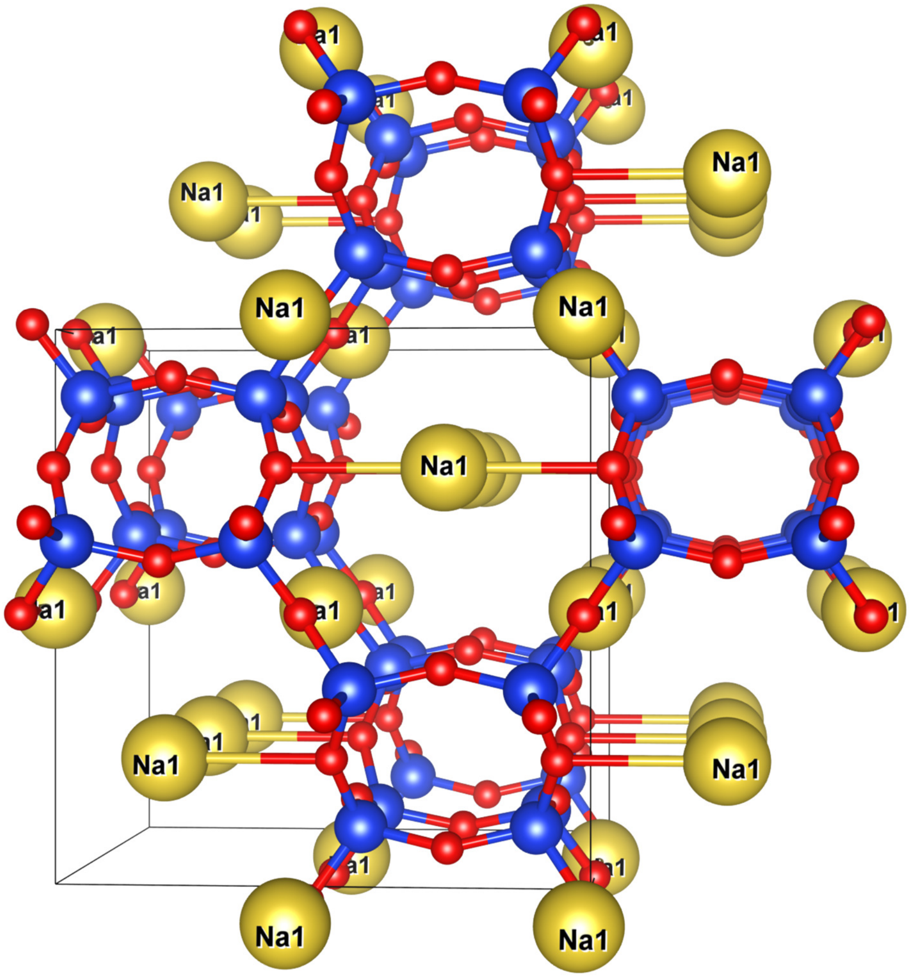

11. Na-GIS (min Si/Al = 1)

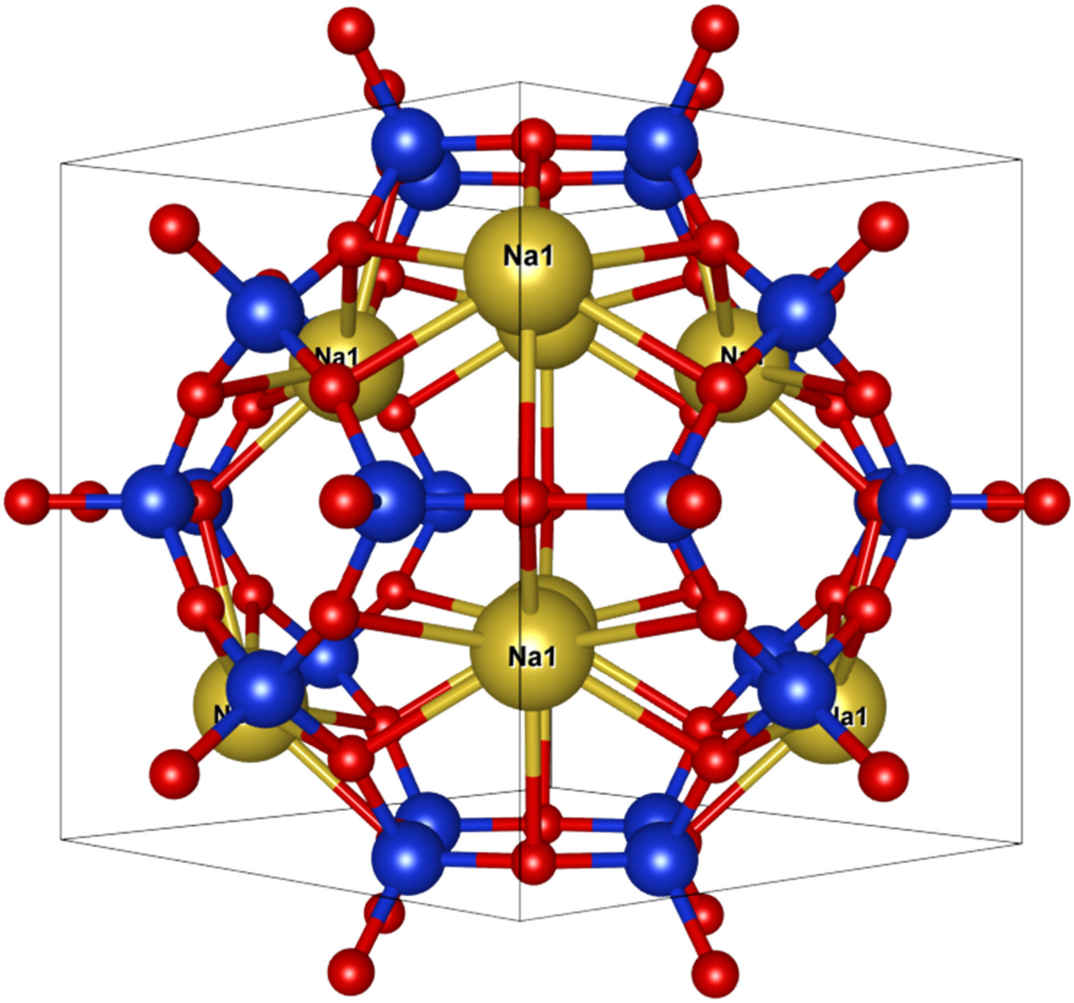

12. Na-SOD (min Si/Al = 0.5*)

* EDI, SOD and CAN topologies allow inclusion of excess cations, and include anion to avoid violation of Lowenstein's rule.

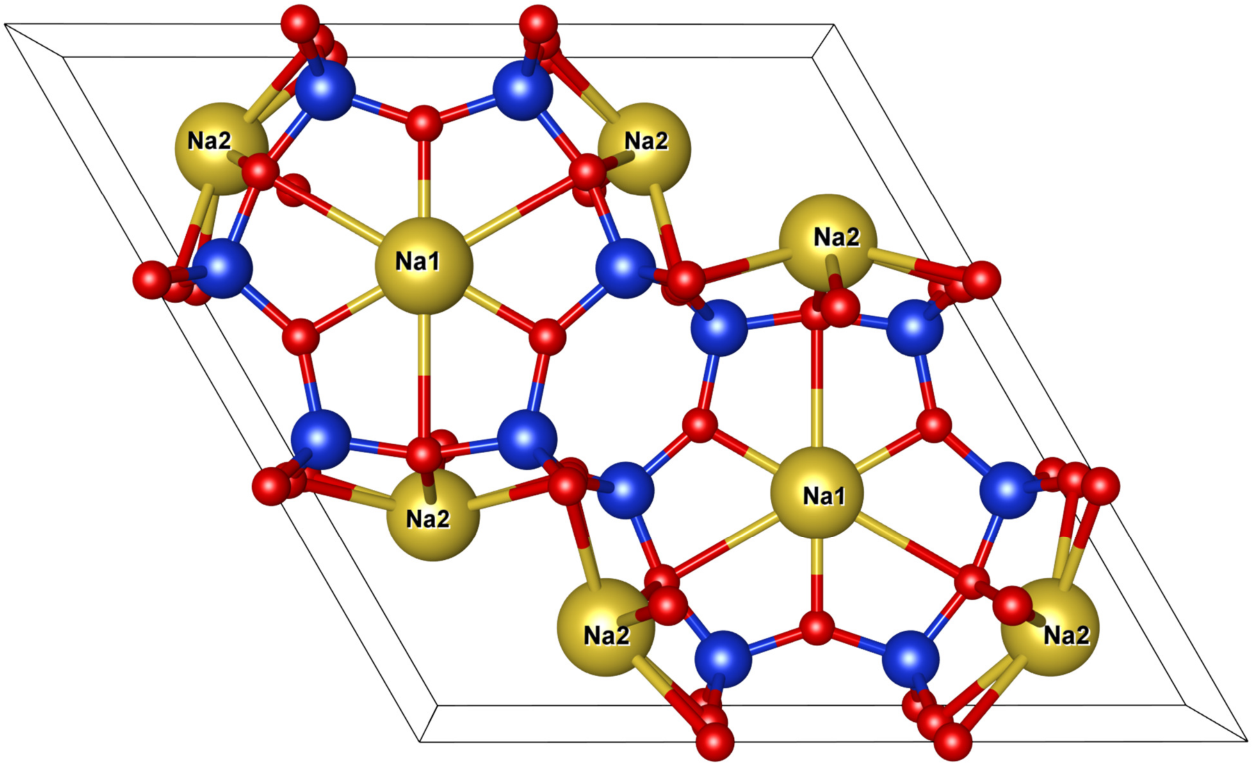

13. Na-CAN (min Si/Al = 0.5*)

* EDI, SOD and CAN topologies allow inclusion of excess cations, and include anion to avoid violation of Lowenstein's rule.

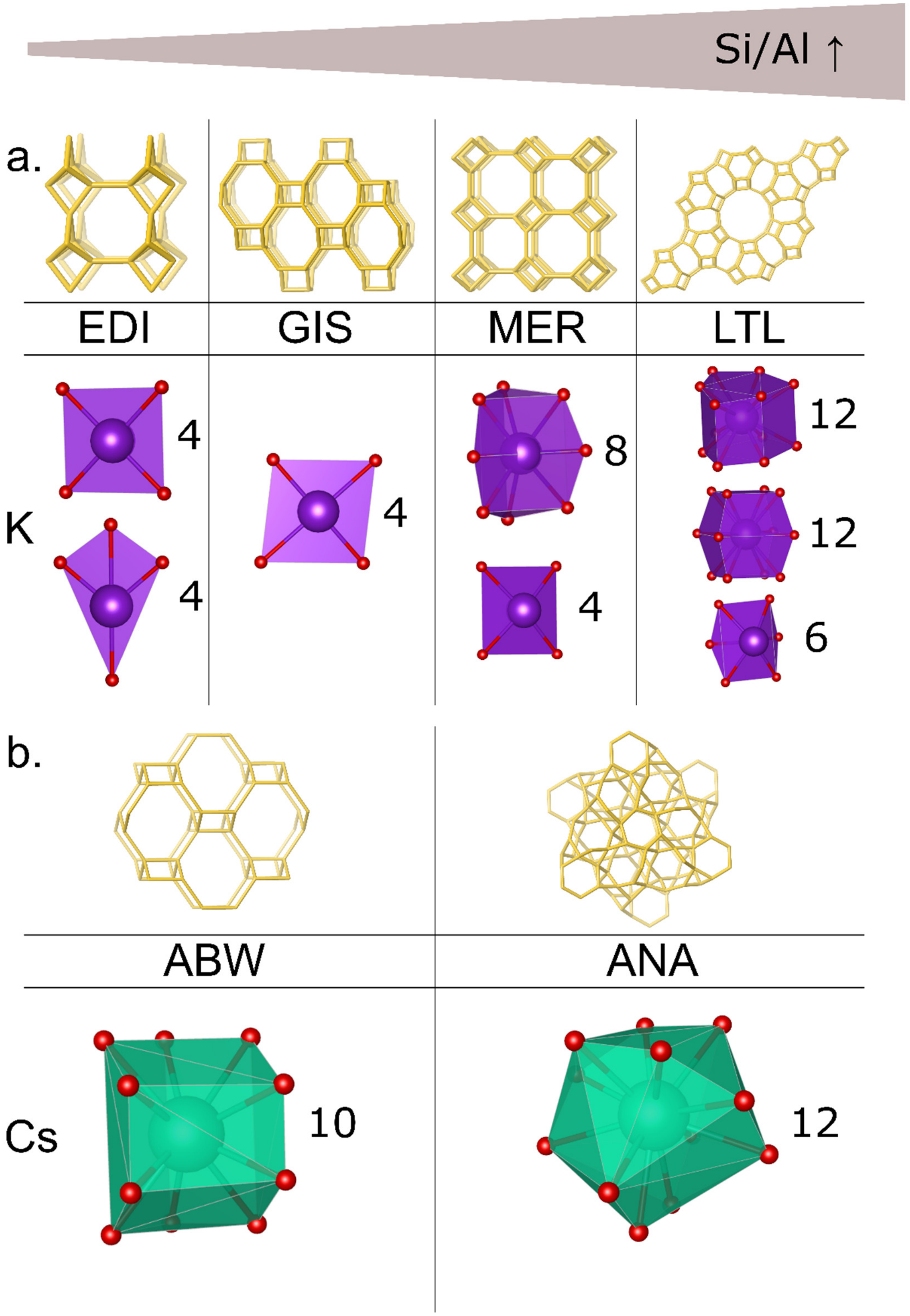

Figure S14: Visualization of cation-framework coordination polyhedral for some potassium (violet) and cesium (green) topologies which form in succession with increasing framework Si/Al ratio. Numbers indicate the coordination number (CN). With increasing framework Si/Al ratio, topologies emerge with cation sites that provide increased CN between framework and cation.

References:

(1) Baerlocher, C.; Xie, D.; McCusker, L. B.; Hwang, S. J.; Chan, I. Y.; Ong, K.; Burton, A. W.; Zones, S. I. Ordered Silicon Vacancies in the Framework Structure of the Zeolite Catalyst SSZ-74. *Nat Mater* **2008**, *7* (8), 631–635.

(2) Ikeda, T.; Inagaki, S.; Hanaoka, T. A.; Kubota, Y. Investigation of Si Atom Migration in the Framework of MSE-Type Zeolite YNU-2. *Journal of Physical Chemistry C* **2010**, *114* (46), 19641–19648.

(3) Momma, K.; Izumi, F. *VESTA 3* for Three-Dimensional Visualization of Crystal, Volumetric and Morphology Data. *J Appl Crystallogr* **2011**, *44* (6), 1272–1276.
